# A Noninvasive BCI System for 2D Cursor Control Using a Spectral-Temporal Long Short-Term Memory Network

**DOI:** 10.3389/fncom.2022.799019

**Published:** 2022-03-23

**Authors:** Kang Pan, Li Li, Lei Zhang, Simeng Li, Zhuokun Yang, Yuzhu Guo

**Affiliations:** Department of Automation Science and Electrical Engineering, Beihang University, Beijing, China

**Keywords:** two-dimensional cursor control, brain-computer interface, electroencephalography, spectral-temporal LSTM network, velocity-constrained loss

## Abstract

Two-dimensional cursor control is an important and challenging problem in the field of electroencephalography (EEG)-based brain computer interfaces (BCIs) applications. However, most BCIs based on categorical outputs are incapable of generating accurate and smooth control trajectories. In this article, a novel EEG decoding framework based on a spectral-temporal long short-term memory (stLSTM) network is proposed to generate control signals in the horizontal and vertical directions for accurate cursor control. Precisely, the spectral information is used to decode the subject's motor imagery intention, and the error-related P300 information is used to detect a deviation in the movement trajectory. The concatenated spectral and temporal features are fed into the stLSTM network and mapped to the velocities in vertical and horizontal directions of the 2D cursor under the velocity-constrained (VC) strategy, which enables the decoding network to fit the velocity in the imaginary direction and simultaneously suppress the velocity in the non-imaginary direction. This proposed framework was validated on a public real BCI control dataset. Results show that compared with the state-of-the-art method, the RMSE of the proposed method in the non-imaginary directions on the testing sets of 2D control tasks is reduced by an average of 63.45%. Besides, the visualization of the actual trajectories distribution of the cursor also demonstrates that the decoupling of velocity is capable of yielding accurate cursor control in complex path tracking tasks and significantly improves the control accuracy.

## 1. Introduction

For a long time, humans have dreamed of controlling external devices through brain activity. The development of brain computer interfaces (BCIs) which aim to directly connect the brain and the external world realized this dream (He et al., 2020). One of the ultimate goals is to decipher the brain activity of patients in real time so that highly dexterous prostheses or exoskeletons can perform anthropomorphic movements. The core task of noninvasive BCIs is to decode real-time (online) brain signals from electroencephalography (EEG) recordings.

Cursor control, designed to map brain signals to the movement of a cursor on a computer screen, is one of the most commonly studied BCI tasks. Cursor control tasks can be used for severely disabled people to engage in brain activity associated with motor imagery and to ultimately control external devices. Due to its ease of implementation, cursor control has certain significance for further improving the quality of life of the disabled.

Electroencephalography-based 1D cursor control has been well studied, where the changes of mu (8–13 *Hz*) or beta (13–28 *Hz*) rhythm during different motor imagery tasks were detected and classified based on event-related desynchronization/synchronization (ERD/ERS) (Blanchard and Blankertz, 2004; Cheng et al., 2004; Fabiani et al., 2004; McFarland and Wolpaw, 2005). Compared with user-machine interaction with limited 1D control, 2D cursor control which decodes the EEG into motions in two directions has a much wider range of applications. Recent study on BCI systems for 2D cursor control can be implemented based on either mu/beta rhythm (Li et al., 2008, 2010a,b; Long et al., 2011) or SSVEP (Beverina et al., 2003; Trejo et al., 2006; Martinez et al., 2007). Study by Edelman et al. (2019); Suma et al. (2020) also have shown that continuous and asynchronous 2D cursor (and robotic arm) control can be achieved with high accuracy using an EEG-source imaging-based approach to decoding motor imagery tasks. Open-loop systems with fixed predefined velocities have also been studied, where different signals such as mu/beta rhythm and P300 are used to control the horizontal and vertical position of the cursor (Wolpaw and McFarland, 2004). These methods can achieve accurate and complex control movements to a certain extent. Furthermore, Wolpaw and McFarland (2004) mapped EEG features to the continuous velocities of the cursor by using a linear regression method, but this decoding model is too simplistic.

The difficulty in 2D cursor control lies in how to correctly decode the motor intention of the operator and control the cursor to move along an expected direction. Movement in an arbitrary direction can be achieved by combining velocities in mutually perpendicular horizontal and vertical directions on a 2D plane. However, the cursor may deviate from the target direction when the directions of decoding velocities are coupled with each other. The deviation from the designed direction can result in serious safety issues and additional power consumption in practical external device control. Therefore, decoding the independent velocity of the two directions is the core of the 2D cursor control.

Two types of signals, namely, active and passive signals are generated during the cursor control tasks. The active signal generated when the user wants to move the cursor to the target is actively generated by the user. The effectiveness of the active signal-based methods often depends on intensive user training (Li et al., 2010a). Passive signal refers to the error signal passively generated in the user's brain when the cursor deviates from the target position but the user does not issue the control intention (Krol et al., 2018). The introduction of the passive signal can alleviate the intensive training of users and form a self-adaptive BCI system to achieve more accurate and complex movements (Krol et al., 2018). The active signal is often measured in the frequency domain as Power Spectral Density (PSD) (Meng et al., 2016), Adaptive Auto Regressive (AAR) model parameters, and wavelet band energy. The corresponding methods include Fast Fourier Transformation (FFT) (Gao et al., 2003), AAR (Schlögl et al., 2005) model, and Wavelet Transform (WT) (Wu and Yao, 2007).

Error-related time domain dynamics which was generated in EEG when an external device does not act as expected are often used passive signal. Many BCI systems operating in an open loop ignore this error signal (Meng et al., 2016; Gao et al., 2017; Chen et al., 2018; Xu et al., 2019). The open-loop BCI control system adjusts the control trajectory by generating extra control signals. This may greatly increase the user's cognitive burden. Even worse, the transient error signal can infiltrate useful signals and disturb the decoding of task related spectrum. Extracting the temporal error information and adaptively correcting the erroneous movements can benefit the BCI control (Rakshit et al., 2020). Commonly used error signals include error-related potentials (ErrP) and P300 (Chavarriaga and Millán, 2010; Chavarriaga et al., 2014). Error-related potentials is a negative deflection of about 250 ms from the medial frontal area after the subject observes a machine (or human) error. P300 is an event-related potential (ERP) when the subjects focus on some important but rare stimulus and the EEG show a positive deflection in about 300 ms. In general, ErrP is a reliable feedback signal. However, ErrP amplitude and latency are also prone to inter-subject and inter-trial variability (Pailing and Segalowitz, 2004; Colino et al., 2017). Additionally, ErrP is best elicited by discrete events (Kumar et al., 2019). For subjects with spinal cord injury and schizophrenia, its magnitude can diminish (Alain et al., 2002; Kerns et al., 2005). Since the movement of the cursor is continuous, ErrP is less suitably used as the feedback marker (Rakshit et al., 2020). In this study, P300 of operators were used as the passive temporal signal to improve cursor movement.

Electroencephalography decoding is to convert brain signals into control signals of the cursor. The output of classifiers such as KNN, LDA (Xu et al., 2019), and SVM (Rakshit et al., 2020) are often used as the control signals. However, classification-based methods generate stepwise cursor movement and are not suitable for continuous complicated operations. Studies have shown that corticospinal excitability during motor imagery is proportional to the motor imaginary intensity (Williams et al., 2012), which makes possible continuous cursor control using the intensity information. Recent study (Meng et al., 2016) mapped the EEG features to the velocity of cursors or robotic arms by using a linear regression method to produce imaginary intensity dependent velocity, which can achieve a certain extent accurate, complicated movements. However, the parameters in the linear model were set subjectively instead of being trained through a supervised learning manner, and the EEG activity from the controlling channels was spatially averaged by a small Laplacian filter.

In this article, a novel EEG decoding framework combining both active and passive EEG signals will be proposed to generate continuously changing velocities. Through decoupling the velocities, the control accuracy of the 2D cursor is able to be significantly improved. Specifically, the proposed method adopts the long short-term memory network (LSTM) backbone (Hochreiter and Schmidhuber, 1997), which is a special recurrent neural network for processing sequence data (Greff et al., 2016). The new framework, called the spectral-temporal LSTM (stLSTM) model, is employed as the EEG decoding model in this article, with the input as the spectral and temporal features and the output as the cursor velocity. The stLSTM enables the model to automatically combine spectral and temporal features rather than average features artificially. In addition, the introduction of the regularization term of velocity-constrained (VC) loss enables the model to fit the velocity in one direction while suppressing the velocity in the other direction when training the control signals in the horizontal and vertical directions at the same time, so as to obtain decoupled control signals.

The main contributions of this study can be summarized as follows: (1) Error-related temporal features P300 are utilized to detect a deviation in the movement trajectory; (2) The spectral and temporal features are used as the input of the decoding model, which can describe the EEG signal more comprehensively; (3) The LSTM network is used as the decoding model to generate control velocities due to its good ability to extract the contextual relationship of the time series; (4) A VC loss is introduced to fit the velocity in the imaginary direction and suppress the velocity in the non-imaginary direction to further optimize the trajectory control of the 2D cursor.

## 2. Real BCI Control EEG Dataset Acquisition

### 2.1. BCI Control Dataset and Preprocessing

The evaluation of the performance of our study is based on the public EEG dataset (which is available online at https://doi.org/10.5061/dryad.nh109). The experiments were conducted at the University of Minnesota, and the EEG data were recorded using a 64-channel Neuroscan cap with SynAmps RT headbox and SynAmps RT amplifier (Neuroscan Inc, Charlotte, NC). The EEG signals were sampled at a rate of 1,000 Hz and bandpass-filtered in the range of 0.5–200 Hz. A notch filter of 60 Hz was applied to the raw EEG signals to remove the power line interference. Refer to Meng et al. (2016) for the detailed experimental settings.

Motor imagery tasks were used to control and drive a 2D virtual cursor or a robotic arm to move. Left, right, two-handed and relaxed motor imagery corresponds to the left, right, up, and down movement of the 2D virtual cursor or the robotic arm, respectively. The dataset contains data for five tasks: the virtual-cursor-only task, which is a unidirectional motor imagery task without visual feedback, including experiments named 1DPreRun and 2DPreRun; the other four are reach-and-grasp tasks with visual feedback.

Data from a total number of 10 EEG channels around C3 and C4 in the left and right motor cortex were utilized for the control of cursor movement as in Meng et al. (2016), including channels in the fronto-central areas that contribute most to the error-related signal (Chavarriaga and Millán, 2010). The specific channels were: C1 C2 C3 C4 C5 C6 FC3 FC4 CP3 CP4 as in the standard 10–20 system. In order to increase the calculation speed and hardly affect model performance, the signals were down-sampled to 100 *Hz*. Then a 0.1–30 *Hz* band-pass filter was used to remove extraneous information in the signals.

### 2.2. EEG Dataset Settings

The proposed stLSTM model is trained in a supervised learning manner. In this study, BCI control EEG data in the 2DPreRun task from 13 subjects are used to train the model, and data from other tasks are used to evaluate the model. The data of each subject in the 2DPreRun task is divided into a training set (TRAIN) and a testing set (TEST) by the proportion of 7:3, respectively, where the number of samples of the four categories is balanced. Furthermore, the testing set TEST is divided into the TESTUD set (only UD samples are included) and the TESTLR set (only LR samples are included). The velocity labels for supervised training are defined as follows:

- horizontal velocity: for *x*_*i*_∈{*U, D*}, the velocity label is set to 0; for *x*_*i*_∈{*L, R*}, the velocity label is set to the value in the dataset.

- vertical velocity: for *x*_*i*_∈{*L, R*}, the velocity label is set to 0; for *x*_*i*_∈{*U, D*}, the velocity label is set to the value in the dataset.

## 3. Methodology

In this section, the notations used in this article are first defined. The framework of the proposed method is then presented, and the modules in stLSTM including spectral features extraction, error-related temporal features extraction, and LSTM with VC loss are discussed in detail. Finally, the pseudo code of the new EEG decoding framework for 2D cursor control is presented.

### 3.1. Definitions and Notations

Assume that the preprocessed EEG signal in each trial is $X_{EEG}\in R^{{}^{n\times m}}$ with *m* channels and *n* sampling points at a sampling rate of *f*_*o*_ Hz. After extracting features from the EEG signal *X*_*EEG*_, segment the feature signal to yield the dataset *D* = {(*x*_1_, *y*_1_), (*x*_2_, *y*_2_), …, (*x*_*N*_, *y*_*N*_)}, where *N* is the total number of samples. Each sample $x_{i}\in R^{{}^{E\times T}}$ is a segment of length *T* along the time dimension, with the *E* is the feature dimension, i.e., the spatial dimension related to the number of channels; $y_{i}\in R^{{}^{1\times 2}}$ represents the velocities in the horizontal and vertical directions of the cursor.

### 3.2. The Framework of stLSTM

**Figure 1:**
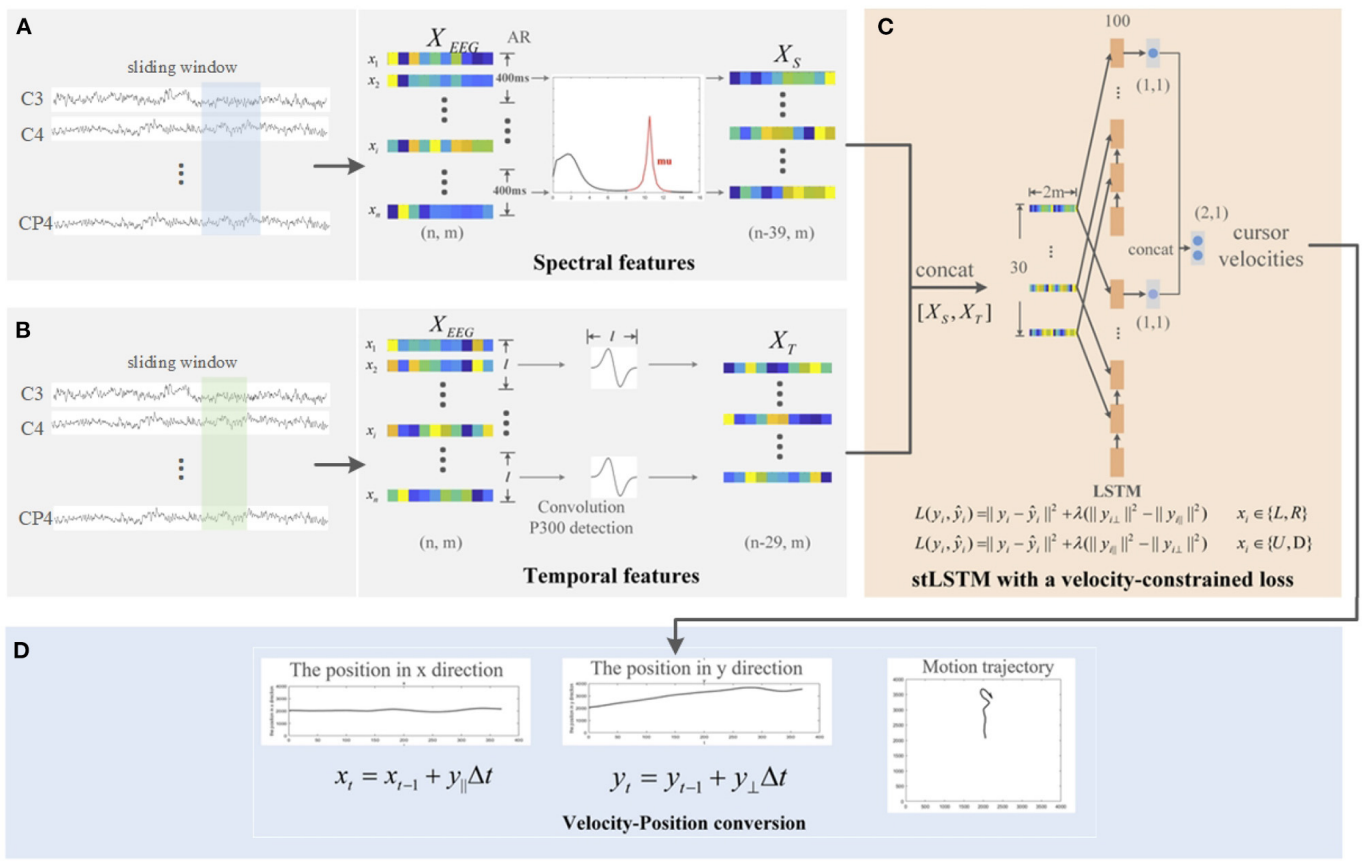
The framework of the proposed spectral-temporal LSTM (stLSTM) method. **(A)** spectrum analysis for spectral features extraction; **(B)** time process analysis for error-related temporal features extraction; **(C)** Long short-term memory (LSTM) network with the velocity-constrained (VC) loss for mapping the input features to the velocities in horizontal and vertical direction; **(D)** integrating the velocities to obtain the movement trajectory of the cursor.

The stLSTM framework proposed in this article is shown in Figure 1. Specifically, the spectrum analysis in Figure 1A extracts the spectral features of the EEG signal, and the time process analysis in (B) extracts the error-related temporal features. The LSTM network with VC loss shown in (C) maps the concatenated spectral and temporal features to the velocity of the cursor in the horizontal and vertical directions. Finally, the velocities in the two directions are, respectively, integrated to obtain the movement trajectory of the cursor, as shown in (D). Therein, in the spectrum analysis and the time process analysis, sliding windows with lengths of 400 *ms* and 300 *ms*, and steps of 10 *ms* are used, respectively. The specific computation details will be discussed in subsequent subsections.

### 3.3. Spectral Feature Extraction

The active signal is often measured in the frequency domain. Commonly used power spectrum estimation methods include parametric and nonparametric methods. The autoregressive (AR) method which models the observation generative process with a small number of parameters, can effectively identify the frequency peaks in the spectrum. In this article, the AR method is adopted to estimate the principal component power spectrum.

An AR model which estimates the amplitudes of *X*_*EEG*_ can be described as:


(1) $$\begin{array}{r} X_{EEG_{j}}\left(t\right)=\overset{p}{\underset{i=1}{\sum }}w_{j}\left(t-i\right)X_{EEG_{j}}\left(t-i\right)+\epsilon \end{array}$$

where *X*_*EE*_*G*__*j*__(*t*) is the estimated signal of the *j*-th channel at time *t*, *p* is the order, *w*_*j*_(*t*) is the coefficient, and ϵ is the white noise in the AR model. The coefficients are estimated by the Burg algorithm. In the current study, we applied the 16th order AR model with a window length of 400 *ms* and a step length of 10 *ms* as in Meng et al. (2016).

According to the relationship between the power spectrum and the frequency response function, the power spectrum density *P* can then be calculated as follows:


(2) $$\begin{array}{r} P_{j}\left(e^{iw}\right)=\frac{\sigma^{2}}{|1+\sum_{k=1}^{p}w_{j}\left(k\right)e^{-iwk}{|}^{2}} \end{array}$$

where σ^2^ is the variance of the white noise sequence. Previous studies (Meng et al., 2016; Paek et al., 2019; Rakshit et al., 2020) have shown that in the motor imagery paradigm, subjects experience significant ERD/ERS phenomena, which often appear in the mu (8–13 *Hz*) frequency band. Therefore, the energy in mu rhythm which is the sum of the area under the PSD in the mu band will be used as the spectral feature.


(3) $$\begin{array}{r} X_{S_{j}}\left(t\right)=\underset{f\in 8-13Hz}{\sum }P_{j}\left(f\right)\Delta f \end{array}$$

where *f* is the frequency and Δ*f* is the interval between two adjacent frequencies. Hence, the spectral feature $X_{S}\in R^{{}^{\left(n-39\right)\times m}}$ is obtained as shown in Figure 1A.

Moving the window yields the sequences of the mu band energy of each principle component. These spectral features represent uncorrelated decoupled mu band energy corresponding to each of the eigen-brains.

### 3.4. Error-Related Temporal Feature Extraction

For an open-loop BCI control system, the real trajectory may not agree well with the expected movement. When the trajectory deviates from the ideal trajectory, the model proposed in this article will utilize the error-related P300 signals to detect this deviation and make the current trajectory tend to the ideal trajectory. Therefore, error-related temporal signals can alleviate safety problems caused by unexpected movement of the cursor, thus, equipping the BCI systems with a better control performance.

In addition to the frequency domain features, error-related temporal information is also taken into account to form a more adaptive BCI system. When the subjects notice that the cursor does not reach the expected control position, a positive potential, known as P300, is generated in the brain at about 300ms after the stimulation (Rakshit et al., 2020). The time domain features should capture the time-varying characteristics of the amplitude of the ERP, such as P300. In this study, the time domain features are defined as follows (Yeom et al., 2013):


(4) $$\begin{array}{r} X_{T_{j}}\left(t\right)=\frac{1}{l}\overset{i=l-1}{\underset{i=0}{\sum }}\varphi \left(l-i\right)X_{EEG_{j}}\left(t-i\right) \end{array}$$

where *X*_*T*_*j*__(*t*) is the dynamic feature of the *j*-th channel at time *t*, φ(·) is the first-order derivative of the Gaussian wavelet function (Guo et al., 2016), *l* is the scale of φ(·). In the current study, *l* is 30, i.e., the window length is 300 *ms* to obtain dynamic features with a step length of 10 *ms*. Therefore, the dynamic feature at time *t* is the result of the convolution of the wavelet function with the signal in the past 300 ms window, and error-related temporal feature $X_{T}\in R^{{}^{\left(n-l+1\right)\times m}}$ was obtained as in Figure 1B.

Before being fed into the stLSTM network, the *X*_*S*_ and *X*_*T*_ feature vectors are first right-aligned along the time dimension, and the features of the first 10 redundant time steps of *X*_*T*_ are removed. Then concatenate the feature vectors *X*_*S*_ and *X*_*T*_ to obtain the final spectral-temporal feature vector:


(5) $$\begin{array}{r} X=\left[X_{S},X_{T}\right] \end{array}$$

where *X*∈*R*^(*n*−39) ×2*m*^ is the final feature vector for a trial. Then segmenting this feature signal yields the dataset *D* = {(*x*_1_, *y*_1_), (*x*_2_, *y*_2_), …, (*x*_*N*_, *y*_*N*_)}, where $x_{i}\in R^{{}^{E\times T}}$ is a sample with *E* = 2*m* and sequence length *T*, *N* is the total number of samples in a trial. In this study, we take *T* = 30, so there is *N* = (*n*−39)/30 for each trial.

### 3.5. stLSTM With VC Loss

Electroencephalography decoding can be considered as a time series recognition task. LSTM can effectively capture the contextual dependency of time series, which is very suitable for this task. A new stLSTM with spectral and error-related temporal features will be used for the EEG decoding in the 2D cursor control tasks.

The stLSTM maps the concatenated feature vector X to the velocities in the horizontal and vertical direction. In order to improve the control accuracy in the two decoupled directions, a new velocity-constrained (VC) is introduced to fit the velocity in one direction and suppress the velocity in the orthogonal direction. The basic mean squared error (MSE) loss function of LSTM is defined as follows:


(6) $$\begin{array}{r} L_{MSE}\left(y_{i},\hat{y_{i}}\right)=||y_{i}-\hat{y_{i}}|{|}^{2} \end{array}$$

where *y*_*i*_ denotes the velocities and $\hat{y_{i}}$ denotes the model prediction.

Introducing the repellent constraints, the proposed VC loss is defined as follows:


(7) $$\begin{array}{r} \begin{array}{c} L_{VC}\left(y_{i},\hat{y_{i}}\right)=||\hat{y_{i⊥}}|{|}^{2}-||\hat{y_{i∥}}|{|}^{2}\;X_{i}\in \left\{L,R\right\}\\ L_{VC}\left(y_{i},\hat{y_{i}}\right)=||\hat{y_{i∥}}|{|}^{2}-||\hat{y_{i⊥}}|{|}^{2}\;X_{i}\in \left\{U,D\right\} \end{array} \end{array}$$

where $\hat{y_{i⊥}}$ denotes velocity in the vertical direction and $\hat{y_{i∥}}$ denotes velocity in the horizontal direction, {*L, R*} and {*U, D*} are the datasets of horizontal and vertical motion, respectively.

Spectral-temporal LSTM imposes control of output velocities on this basis and punishes wrong movements, with the final loss function as follows:


(8) $$\begin{array}{r} L_{stLSTM}\left(y_{i},\hat{y_{i}}\right)=L_{MSE}\left(y_{i},\hat{y_{i}}\right)+\lambda L_{VC}\left(y_{i},\hat{y_{i}}\right) \end{array}$$

where λ is the regularization parameter, which affects the velocity control. For *x*_*i*_∈{*L, R*}, stLSTM will suppress the vertical velocity and fit horizontal velocity; for *x*_*i*_∈{*U, D*}, stLSTM will suppress the horizontal velocity and fit vertical velocity.

Shown as Figure 1C, the net model is designed with four layers: input layer, LSTM layer, Fully Connected (FC) layer, and output layer. Specifically, the size of each input sample is 30 × 2*m*, where 30 is the sequence length and 2*m* is the number of features at each time step. In this study, the number of hidden neural units in the LSTM layer is 100, and the output of the stLSTM is the horizontal and vertical velocity of the cursor. The input data is passed through two LSTM layers and an FC layer to predict the velocity in the corresponding direction separately, and the output data is concatenated together in the output layer as the final velocity.

### 3.6. stLSTM Algorithm

The pseudo code of the stLSTM pipeline is described in Algorithm 1 where the input terms include EEG signal (*X*_*EEG*_), number of training epochs (*T*), and the candidates for regularization parameter (λ_*list*_). Precisely, 0.01:0.01:0.1 indicates λ varies from 0.01 to 0.1 in step of 0.01. For each λ, it starts with initializing *stLSTM*_λ_; the optimizer is adam, and batch gradient descent (GD) is used to train the model until the number of epochs is reached. Finally, the best model *stLSTM*_*best*_ is selected according to the *MSE*_*best*_.

**Algorithm 1:** Spectral-temporal LSTM with a velocity-constrained loss

Input: EEG signal *X*_*EEG*_, training epochs *T*, the candidates for regularization parameter λ_*list*_ = [0.0001, 0.001:0.001:0.09, 0.01:0.01:0.1];
Output: The best stLSTM model *stLSTM*_*best*_
1: Extracting *X*_*S*_ based Equation 1 ~ Eq.3;
2: Extracting *X*_*T*_ based Equation 4;
3: Generating training dataset *D* and testing dataset *P* based on Eq.5;
4: λ_*best*_ = 0.0001
5: *MSE*_*best*_ = *inf*
6: *stLSTM*_*best*_ = *stLSTM*(*init*)
7: if λ = λ_*list*_ **then**
8: Initializing parameters in the *stLSTM*_λ_ as Θ^(0)^;
9: *t* = 0;
10: while *t* < *T* **do**
11: Calculating feedforward output ${Ŷ}^{\left(t\right)}=stLSTM_{\lambda }\left(D,\Theta^{\left(t\right)}\right)$;
12: Calculating *loss*^(*t*)^ based on Eq.8;
13: Calculating gradient *dΘ*^(*t*)^;
14: Updating parameter Θ^(*t*+1)^ = *adam*(Θ^(*t*)^, *dΘ*^(*t*)^);
15: ++*t*;
16: end **while**
17: Calculating *MSE*_λ_ based on *stLSTM*_λ_ and testing dataset *P*;
18: if *MSE*_*best*_>*MSE*_λ_ **then**
19: λ_*best*_ = λ
20: *MSE*_*best*_ = *MSE*_λ_
21: *stLSTM*_*best*_ = *stLSTM*_λ_
22: end **if**
23: end **if**

## 4. Experimental Results

This section presents the experimental results of the proposed stLSTM method on the publicly available real BCI control dataset mentioned in Section 2. The performance of the proposed method is compared with the method in Meng et al. (2016), which is obtained by the Linear Regression method using only spectral features (sLR).

Since the nature of the 2D cursor control task is to regress, the root mean square error (RMSE) of the model's predicted velocities is used as the metric to evaluate the performance of the stLSTM and sLR on the testing sets mentioned in Section 2. In order to further illustrate the effectiveness of the spectral features and temporal features, ablation experiments are implemented, in which models using only spectral features (sLSTM) and models using only error-related temporal features (tLSTM) are evaluated.

Notably, the velocities produced by the sLR model in the imaginary direction are used as the target values for the outputs of the model, as defined in Section 2. Hence, the prediction error of the sLR model in the imaginary direction is zero.

### 4.1. The Overall Performance

This section gives the average RMSE results across all 13 subjects of sLR and stLSTM under the *L*_*MSE*_ loss function and *L*_*stLSTM*_ loss function on the TESTUD, TESTLR, and TEST sets, aiming at trying to compare and quantitatively analyze the decoding performance of the proposed method with the sLR method in the imaginary and non-imaginary direction. The average RMSE results of stLSTM and sLR are shown in Tables 1, 2. First, it should be pointed out that, in view of the particularity of the definition of the velocity labels mentioned above, we pay more attention to the predicted velocity on the TESTUD and TESTLR datasets in the non-imaginary direction, such as the horizontal velocity on TESTUD and the vertical velocity on TESTLR.

**Table 1:** The average root mean square error (RMSE) comparison across 13 subjects for horizontal velocity on testing datasets.

**Method**	**TESTUD**		**TESTLR**		**TEST**	
	***L*_*MSE*_**	***L*_*stLSTM*_**	***L*_*MSE*_**	***L*_*stLSTM*_**	***L*_*MSE*_**	***L*_*stLSTM*_**
sLR	23.56 ± 3.71	–	0.0	–	16.86 ± 3.10	–
stLSTM	7.28 ± 1.70	7.19 ± 1.66	27.64 ± 3.82	27.65 ± 3.73	20.10 ± 2.76	20.07 ± 2.72

*On the TESTUD dataset, the smaller the RMSE in the horizontal direction, the better the ability of the model to suppress the velocity in a non-imaginary (i.e., vertical) direction. On the TESTLR dataset, the relatively small horizontal RMSE of the stLSTM indicates the ability of the model to fit the velocity in imaginary (i.e., horizontal) direction. The overall horizontal RMSE on the TEST dataset measures the overall suppression and fitting ability of the models*.

**Table 2:** The average RMSE across 13 subjects comparison for vertical velocity on testing datasets.

**Method**	**TESTUD**		**TESTLR**		**TEST**	
	***L*_*MSE*_**	***L*_*stLSTM*_**	***L*_*MSE*_**	***L*_*stLSTM*_**	***L*_*MSE*_**	***L*_*stLSTM*_**
sLR	0.0	–	23.46 ± 2.74	–	16.44 ± 2.25	–
stLSTM	20.71 ± 1.83	20.69 ± 1.86	8.49 ± 1.35	8.63 ± 1.23	15.97 ± 1.29	16.00 ± 2.25

*On the TESTUD dataset, the smaller the RMSE in the vertical direction, the better the ability of the model to suppress the velocity in a non-imaginary (i.e., horizontal) direction. On the TESTLR dataset, the relatively small vertical RMSE of the spectral-temporal long short-term memory (stLSTM) indicates the ability of the model to fit the velocity in imaginary (i.e., vertical) direction. The overall velocity RMSE on the TEST dataset measures the overall suppression and fitting ability of the models*.

Table 1 shows the RMSE of the horizontal velocity for the two models on the TESTUD, TESTLR, and TEST sets. The optimal λ values of stLSTM under the *L*_*stLSTM*_ loss function are 0.007, 0.01, 0.01, respectively on TESTUD, TESTLR, and TEST set. Therein, it appears that the RMSE of sLR on the TEST dataset is smaller than that of stLSTM, however, the main reason here is the special definition that sLR has vertical RMSE of 0 on TESTUD, which is not our main concern. On the TESTUD dataset, since the ideal horizontal velocity is 0, the RMSE of the horizontal velocity of the proposed method is much smaller than sLR, which indicates that stLSTM effectively suppresses the velocity in the non-imaginary direction. In addition, the RMSE of the horizontal velocity on the TESTLR dataset quantifies the fitting performance of the model's velocity in the imaginary direction. Although the main concern is whether the velocity in the non-imaginary direction is suppressed, it is still expected that the moving direction is able to follow the operator's intention well.

Table 2 shows the RMSE of the vertical velocity for the two models on the TESTUD, TESTLR, and TEST sets. The optimal λ values of stLSTM under the *L*_*stLSTM*_ loss function are 0.08, 0.002, 0.005, respectively on TESTUD, TESTLR, and TEST set. Therein, the RMSE of stLSTM is significantly smaller than that of sLR on the TEST dataset. Similarly, on the TESTLR dataset, since the ideal vertical velocity is 0, the RMSE of the velocity in the vertical direction of the proposed method is significantly smaller than that of sLR, which can indicate that stLSTM effectively suppresses the velocity in the non-imaginary direction. Likewise, the RMSE results on the TESTUD dataset can demonstrate how well the model output can follow the operator's intention in the desired direction.

In order to test the significance of the mean difference in RMSE generated by the sLR and the stLSTM on the testing sets of 13 subjects, a one-way Analysis of Variance (one-way ANOVA) is implemented. Under the condition that the model loss function is *L*_*MSE*_, the one-way ANOVA results showed a significant effect based on the choice of methods (sLR and stLSTM) on the horizontal RMSE on the TEST set (the *F* value of the sum of squares between groups = 7.899; *p* = 0.010), on the horizontal RMSE on TESTUD set (*F* = 206.874; *p* < 0.001), on the vertical RMSE on TESTLR set (*F* = 312.964; *p* < 0.001). While the one-way ANOVA results showed no significant effect on the vertical RMSE on the TEST set (*F* = 0.423; *p* = 0.522) based on the choice of sLR and stLSTM, this makes sense because of the particularity of the definition of the velocity labels mentioned in Section 2.

In general, the RMSE of the proposed method in the non-imaginary directions on the testing sets of the 2D control task is reduced by an average of 63.45%. To sum up, the stLSTM model combining frequency and temporal features can better describe EEG signals and automatically combine spectral and temporal features in an optimal way, hence it has better decoding performance than linear regression models.

In theory, for the loss *L*_*stLSTM*_, the optimization goal of the model is to minimize the summation of the MSE loss and VC Loss, which is a relatively difficult optimization goal. However, according to the results in Tables 1, 2, it can be found that the model with *L*_*stLSTM*_ loss demonstrates a smaller RMSE than that with *L*_*MSE*_ loss. This shows that the introduced VC loss function is able to obtain decoupled and more accurate control signals, therefore, the decoding model unfolds better control performance.

### 4.2. The Performance of Ablation Experiments

In addition to the model structure (LSTM vs. Linear Regression), another significant difference between the method proposed in this article and the method in Meng et al. (2016) is that the stLSTM uses both the spectral and temporal features of the EEG signals to perform motor imagery decoding and output control signals. In order to further analyze and compare the impact of these two types of features and their combination on the model performance, some ablation experiments are necessary to be conducted. This section lists the RMSE results of the model that only uses temporal features (sLSTM), the model that only uses spectral features (tLSTM), and the model that uses both types of features (tLSTM) in imaginary and non-imaginary directions.

The RMSE results of ablation experiments on integrated all subject's data are shown in Table 3. The optimal λ values of sLSTM under the *L*_*stLSTM*_ loss function are 0.001, 0.06, 0.01, and 0.006 respectively on horizontal TESTUD, vertical TESTUD, horizontal TESTLR, and vertical TESTLR set. The optimal λ values of tLSTM are 0.08, 0.0001, 0.07, and 0.0001. The optimal λ values of stLSTM are 0.007, 0.08, 0.01, and 0.002. In Table 3, when the method used is changed from sLSTM to tLSTM, the RMSE in the imaginary direction increases, and the RMSE in the non-imaginary direction decreases. Spectral features are extracted from active signals generated when the user intends to move the cursor to the target position, thus, helping to fit the velocity in the imaginary direction. Passive error signals are generated when the controlled cursor deviates from the target position without the user's intention, and temporal features extracted from these signals help to suppress velocity in non-imaginary directions. From Table 3, stLSTM demonstrates the smallest RMSE in the imaginary direction; tLSTM demonstrates the smallest RMSE in the non-imaginary direction, and stLSTM also performs very small RMSE in the non-imaginary direction. Therefore, it can be shown that the proposed method is able to best fit the velocity in the motion direction while suppressing the velocity in the non-imaginary direction to a certain extent.

**Table 3:** The RMSE comparison of different features for vertical and horizontal velocities on integrated subjects data.

**Method**	**TESTUD**		**TESTLR**	
	**Horizontal**	**Vertical**	**Horizontal**	**Vertical**
sLSTM	7.65	16.71	17.92	7.97
tLSTM	5.60	23.93	24.99	3.98
stLSTM	7.54	16.37	17.42	5.09

*On the TESTUD dataset, the smaller the horizontal RMSE, the better the model's ability to suppress the vertical velocity; the smaller the vertical RMSE, the better the ability to fit the vertical velocity. The same is true for the TESTLR dataset*.

### 4.3. The Performance on 2DPreRun

The previous two sections present the average RMSE results across 13 subjects of the stLSTM on the TESTUD, TESTLR, and TEST sets, which numerically characterize the control performance of the model output in the imaginary and non-imaginary directions from the perspective of overall testing. Another more intuitive way for analysis is to plot the actual trajectories generated by the stLSTM and the sLR. This section demonstrates the trajectories plots of stLSTM and sLR on the 2DPreRun test set. In addition, this section also introduces a new metric called ACC for measuring the control efficiency and a metric called MAR that considers both ACC and velocity in a non-imaginary direction, and the relevant definitions will be elaborated below.

**Figure 2:**
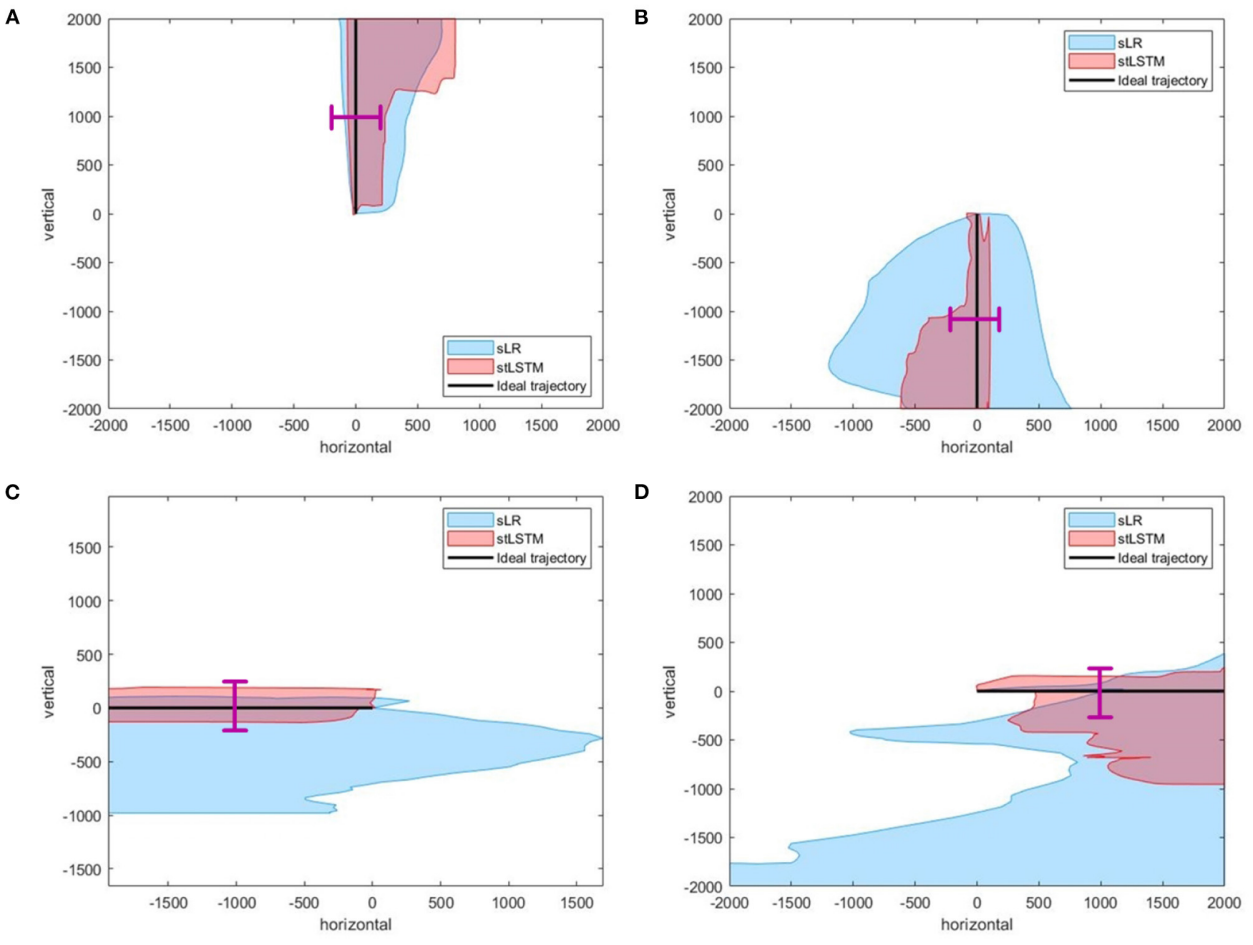
Part of the trajectories. **(A)** up. **(B)** down. **(C)** left. **(D)** right. The black line represents the ideal trajectory of the 2D cursor, the red shadow represents the actual trajectories distribution of the cursor based on stLSTM method, and the blue shadow represents the actual trajectories distribution of the cursor based on sLR method. The magenta lines at the midpoint of the ideal trajectories represent the 400-pixel wide gate mentioned in the Table 5.

The task of 2DPreRun is to control the 2D cursor to reach the target position through motor imagery. The stLSTM is able to output the velocity in the vertical and horizontal direction, and position information can be obtained by integrating the velocities. Part of the trajectories generated by stLSTM and sLR in the 2DPreRun task are shown in Figure 2.

In Figure 2A, the ideal movement trajectory of the 2D cursor is vertically upward. In the process of controlling the upward movement of the cursor, the actual movement direction of the cursor at each moment can be determined and the description of the actual movement direction can be qualitatively measured, which can reflect the control efficiency of the model to a certain extent. One way to define the actual direction at the current moment is the corresponding direction with the highest velocity. The metric ACC is defined as the ratio of the actual number of correct directions to the total number of expected directions. For example, if the ideal direction is upward, the total number of movements is 100, and only 90 of the direction is upward, then the ACC is 90%. Furthermore, when considering the ACC for the expected movement direction, the velocity of the 2D cursor in the non-imaginary direction must also be considered. Therefore, a new metric called root-mean-square-error accuracy ratio (MAR) is introduced here, and the formula is defined as follows:


(9) $$\begin{array}{r} MAR=\frac{RMSE}{\epsilon +ACC} \end{array}$$

where RMSE refers to the RMSE of the velocity in the non-imaginary direction; ACC is the accuracy; ϵ is the smoothing factor to avoid division of zero, set to the same empirical value of 10*e*−8 as adam (Kingma and Ba, 2014). The smaller the value of MAR, the better the performance the model exhibits. From Equation 9, MAR considers both the actual movement direction and the velocity in the non-imaginary direction; since the range of ACC is 0 1, the interval of MAR is larger than that of RMSE, which will produce more precise errors. Therefore, the definition of the metric MAR is persuasive.

The MAR results on TESTUD and TESTLR datasets are shown in Table 4. It can be observed that stLSTM exhibits a smaller MAR compared to sLR, which further illustrates the excellent performance of the proposed method.

**Table 4:** The MAR comparison for velocity in non-imaginary direction on testing datasets.

**Method**	**TESTUD**			**TESTLR**		
	**ACC(*%*)**	**RMSE**	**MAR**	**ACC(*%*)**	**RMSE**	**MAR**
sLR	59.60	17.62	0.84	58.10	22.06	1.02
stLSTM	61.40	5.62	0.09	60.20	8.90	0.11

*The larger the ACC ∈[0−100%], the larger the MAR ∈[0−1], and the smaller the RMSE, indicate that the better the ability of the model to suppress the velocity in a non-imaginary direction*.

To further quantitatively compare the differences in control accuracy between the sLR and the stLSTM, we design the following experiments: for each trial, set a 400-pixel wide gate at the midpoint of the line from the starting point of the movement to the target point, and define that when the actual trajectory of the controlled cursor falls on the outside of the gate then this trial fails, otherwise, it succeeds. We conducted experimental statistics on the imaginary directions of up, down, left, and right on the 2DPreRun dataset. The success rates of all trials of sLR and stLSTM are shown in Table 5. The statistical results show that the control accuracy of the proposed method is higher than that of sLR in all four motor imaginary directions, i.e., more trials can pass through the gate with a width of 400 pixels.

**Table 5:** The comparison for control accuracy.

**Method**	**The imaginary direction**			
	**Up**	**Down**	**Left**	**Right**
sLR	87.50%	62.50%	75.00%	56.25%
stLSTM	93.75%	93.75%	100.00%	81.25%

*For each trial, set a 400-pixel wide gate at the midpoint of the line from the starting point of the movement to the target point, and define that when the actual trajectory of the controlled cursor falls on the outside of the gate then this trial fails, otherwise, it succeeds*.

**Figure 3:**
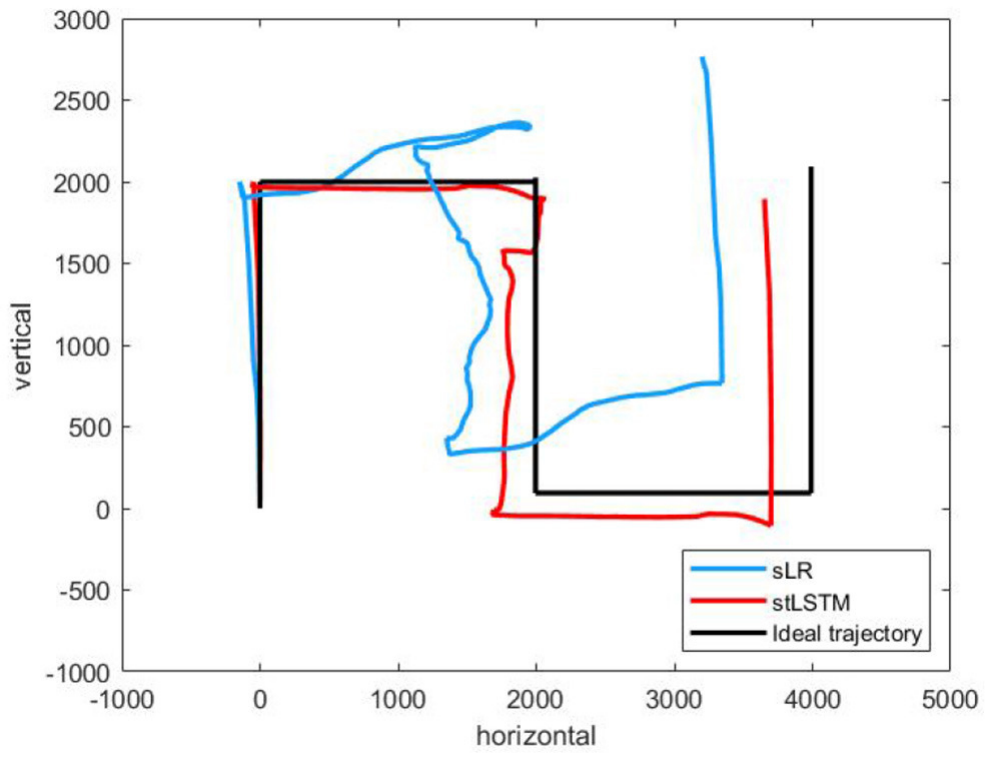
A complex trajectories {up, right, down, right, up}.The black line represents the ideal trajectory of the cursor, the red line represents the trajectory of the cursor based on the stLSTM method, and the blue line represents the trajectory of the cursor based on the sLR method.

Figure 3 presents complex trajectories, which is to control the 2D cursor to move along the directions of {up, right, down, right, up}. The 2D cursor is able to complete complex motion operations more accurately to a certain extent based on the proposed method.

### 4.4. The Performance on Other Tasks

The proposed model was also evaluated on datasets of the four-target grasp task and five-target grasp task. The four-target grasp task refers to that there are four targets in a square area in the real world, which are located up, down, left, and right, respectively, and the subjects performed robotic arm control while a cursor was simultaneously controlled to move on a computer monitor. The five-target grasp task is based on the four-target grasp task with an additional target placed in the center of the square area, and the fifth target is surrounded by the other four targets. Besides, the four-target and five-target grasp tasks differ from the experimental paradigm of the 2DPreRun task mentioned above. In the 2DPreRun task, the motor imaginary signals generated by the subjects to control the movement of the cursor remain consistent throughout the entire trial. For example, if the target is on the right side, the subjects have to implement the imagination of the right hand all the time. In the four-target and five-target grasp tasks, the subjects can choose to generate motor imaginary signals in any direction. For example, when the target is on the right, if the trajectory of the cursor movement starts to shift upward, subjects can implement the imagination of relaxation to control the cursor to move downward. This section presents the trajectory plots of stLSTM and sLR on the four-target grasp and five-target grasp test sets, respectively.

**Figure 4:**
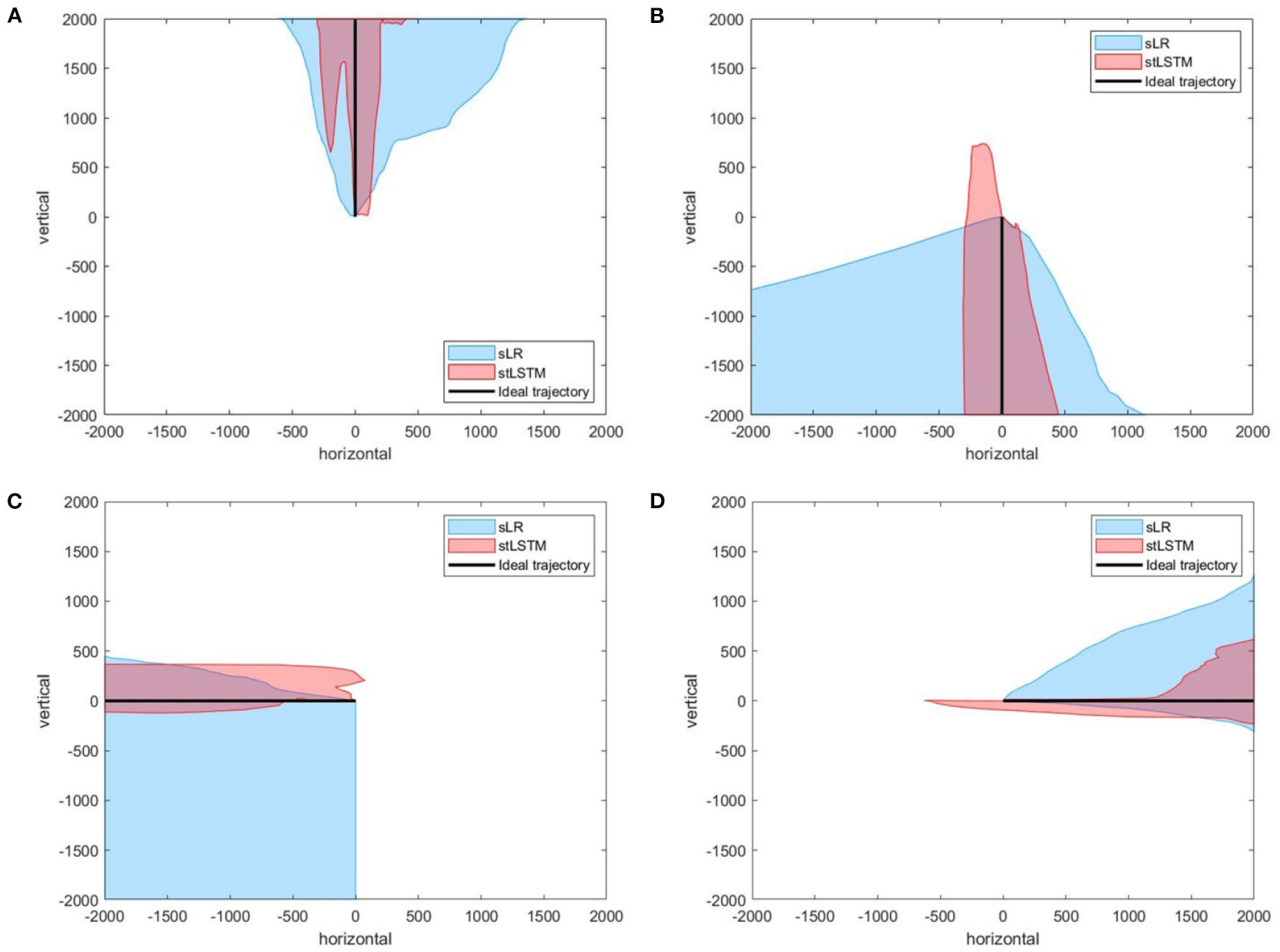
Part of the trajectories distribution of the four-target grasp task. **(A)** up. **(B)** down. **(C)** left. **(D)** right. The black line represents the ideal trajectory of the 2D cursor, the red shadow represents the trajectories distribution of the 2D cursor based on the stLSTM method, and the blue shadow represents the trajectories distribution of the 2D cursor based on the sLR method.

Figure 4 presents the actual trajectories plots of the four-target grasp task. In Figures 4B,D, although the cursor may move in the wrong direction at the beginning, it can be corrected quickly, indicating that stLSTM possesses the ability to effectively correct the trajectory bias. The boundary of the actual trajectories distribution produced by stLSTM is narrower than that of sLR, indicating that the proposed method is equipped with higher control accuracy.

**Figure 5:**
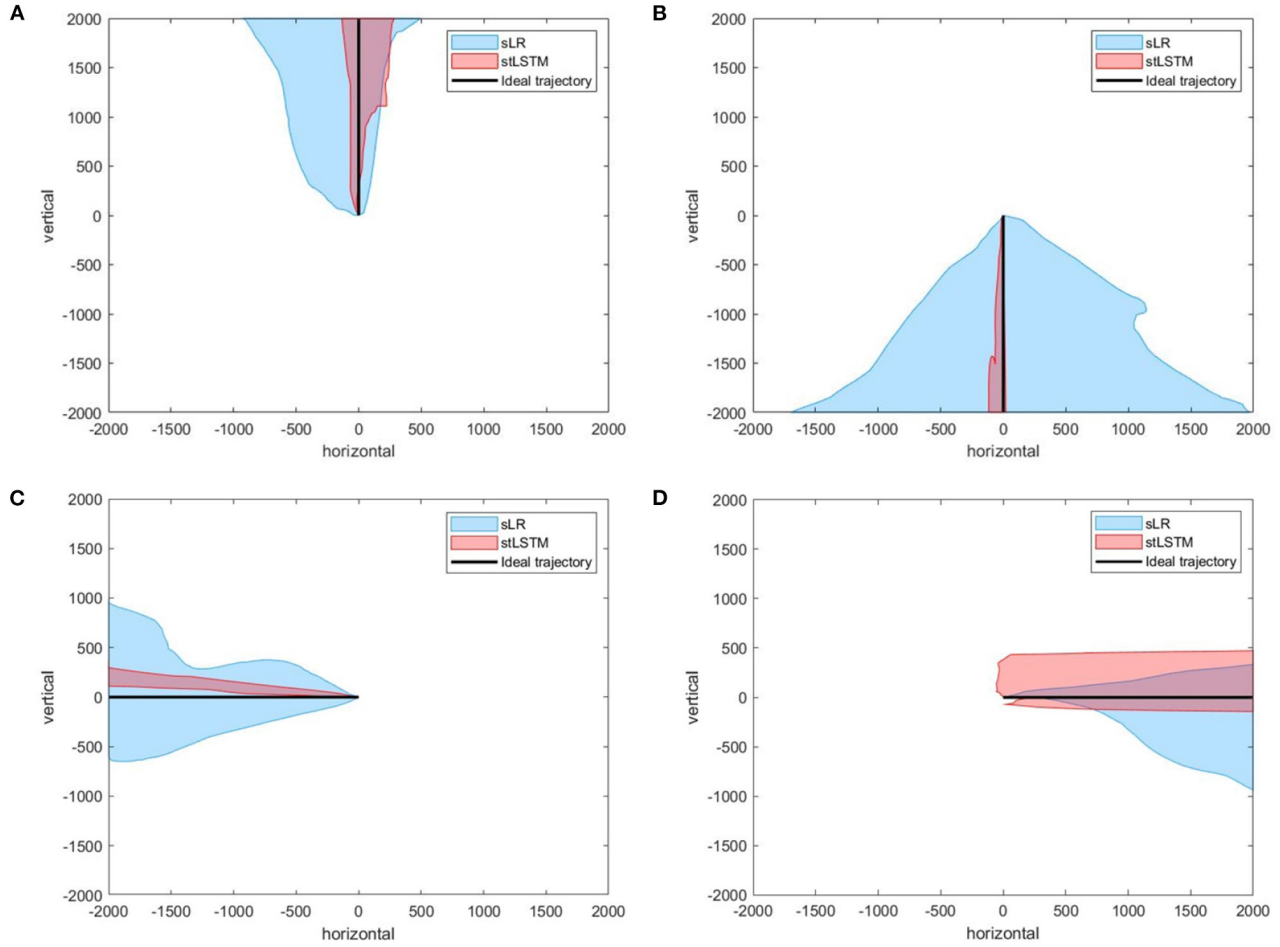
Part of the trajectories of the five-target grasp task. **(A)** up. **(B)** down. **(C)** left. **(D)** right. The black line represents the ideal trajectory of the 2D cursor, the red shadow represents the trajectories distribution of the 2D cursor generated by stLSTM method, and the blue shadow represents the trajectories distribution of the 2D cursor generated by sLR method.

**Figure 6:**
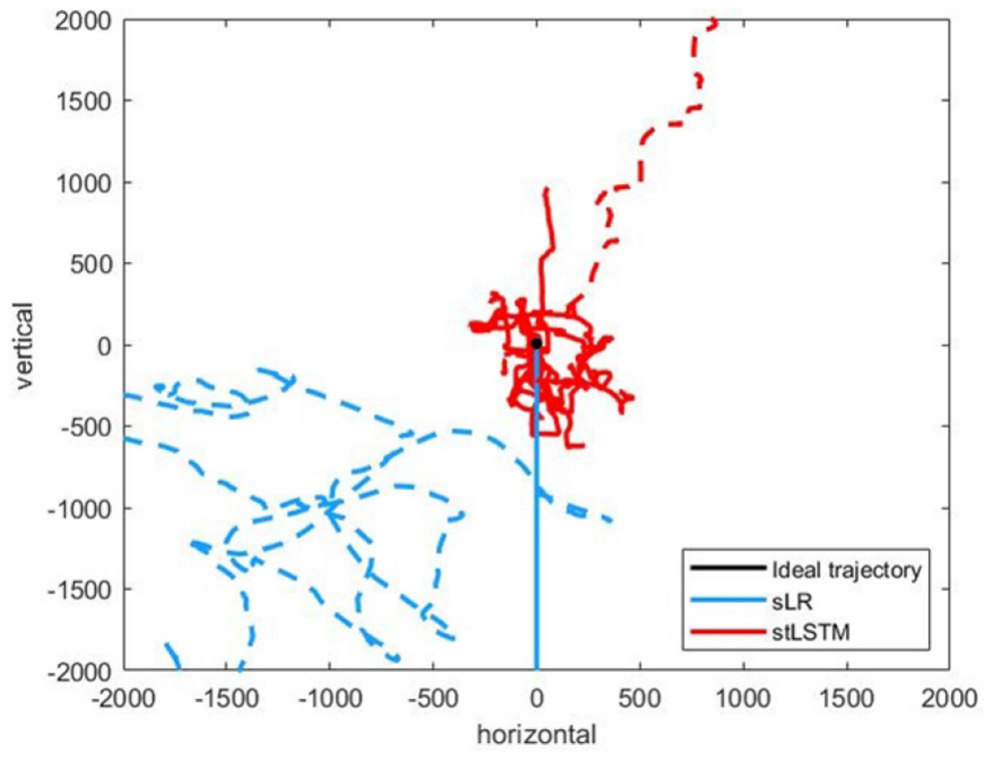
Part of the trajectories of the five-target grasp task. The black dot at the origin represents the ideal trajectory of the cursor hovering over the target, the red line represents the trajectories of the cursor generated by the stLSTM method, and the blue line represents the trajectories of the cursor generated by sLR method.

Figure 5 presents the experimental results of the five-target grasp task. In Figures 5A–C, the boundary of the trajectories distribution of the stLSTM is smaller than that of sLR, which indicates the effectiveness of the proposed method in terms of the accuracy of the control. In Figure 5D, although the error in the positive y-axis of the trajectory set generated by stLSTM is large, its boundary is still smaller than that of sLR, and the error range of the actual cursor movement is stable. In Figure 6, the trajectories generated by stLSTM are basically near to the origin and are not significantly worse than sLR. The dotted line in Figure 6 indicates the special case of divergent trajectories. It is worth pointing out that the stLSTM model trained on 2DPreRun datasets was used to directly evaluate prediction performance on other tasks, which suggests that the proposed method is knowledge-transferable and is able to automatically learn brain pattern-related and underlying features for different downstream tasks.

## 5. Discussions

### 5.1. The Role of stLSTM

The stLSTM possesses the ability to automatically combine spectral and temporal features by optimizing the *L*_*stLSTM*_ loss function rather than average features of the selected channels by a small Laplacian filter. The parameters of the model are updated based on the GD method, and the optimal parameters are learned from the distribution of data, rather than being artificially subjective set. The *L*_*stLSTM*_ loss function is able to train the model to output control signals in both horizontal and vertical directions at the same time to obtain decoupled velocities.

**Figure 7:**
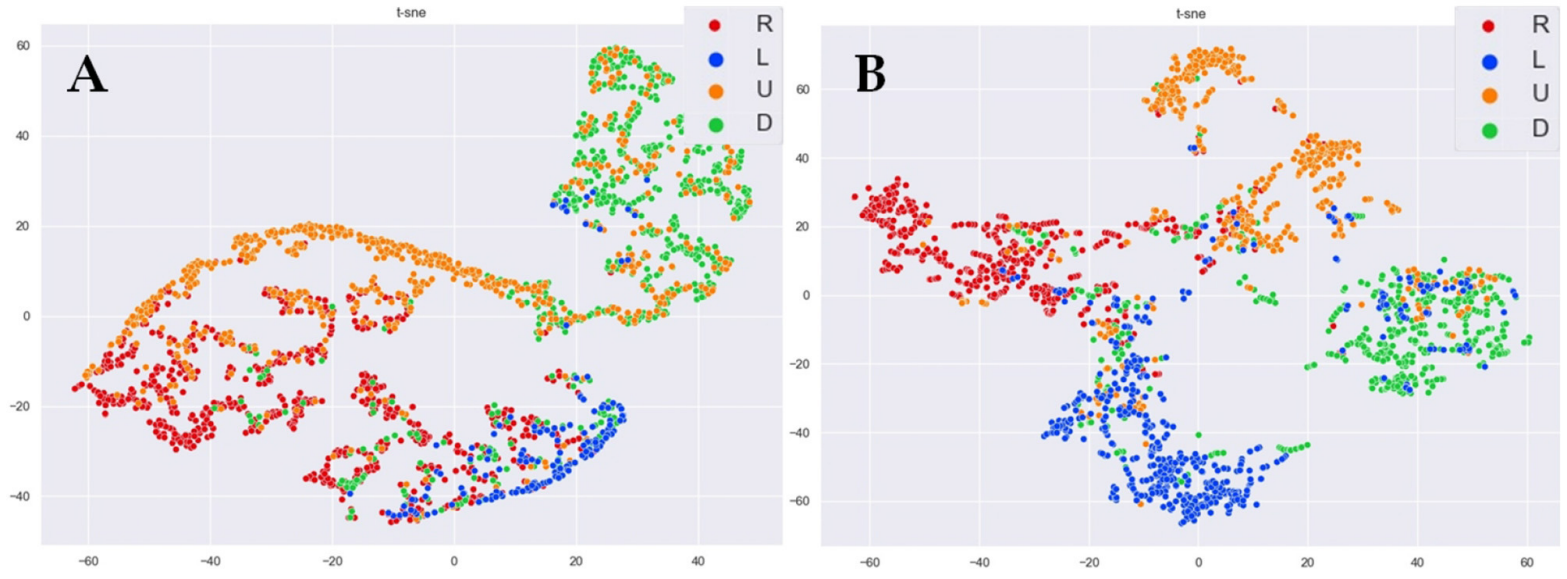
The t-SNE visualization of the features in right, left, up, and down directions. **(A)** sLR. **(B)** stLSTM.

In Figure 7, we adopt the t-distributed stochastic neighbor embedding (t-SNE) (Van der Maaten and Hinton, 2008) algorithm for dimensionality reduction and visualization of the features extracted by sLR and stLSTM. The t-SNE technique visualizes high-dimensional data by giving each datapoint a location in a two or three-dimensional map. From the results in Figure 7, it is noticeable that the features extracted by the LSTM layer in the stLSTM model are visualized as four categories with significantly good distribution discrimination, however, the optimization task of stLSTM is not classification, but regression. As can be seen from Figure 7, compared with the sLR method, stLSTM can still distinguish samples with different target directions, which indicates that stLSTM is able to extract information related to the imaginary direction in brain patterns. These well-discriminating features, rather than the spectral and temporal features initially fed into the model, are linearly mapped to the final 2D cursor velocities through the FC layer, which can effectively improve model performance.

### 5.2. The Role of Error-Related Temporal Features

**Figure 8:**
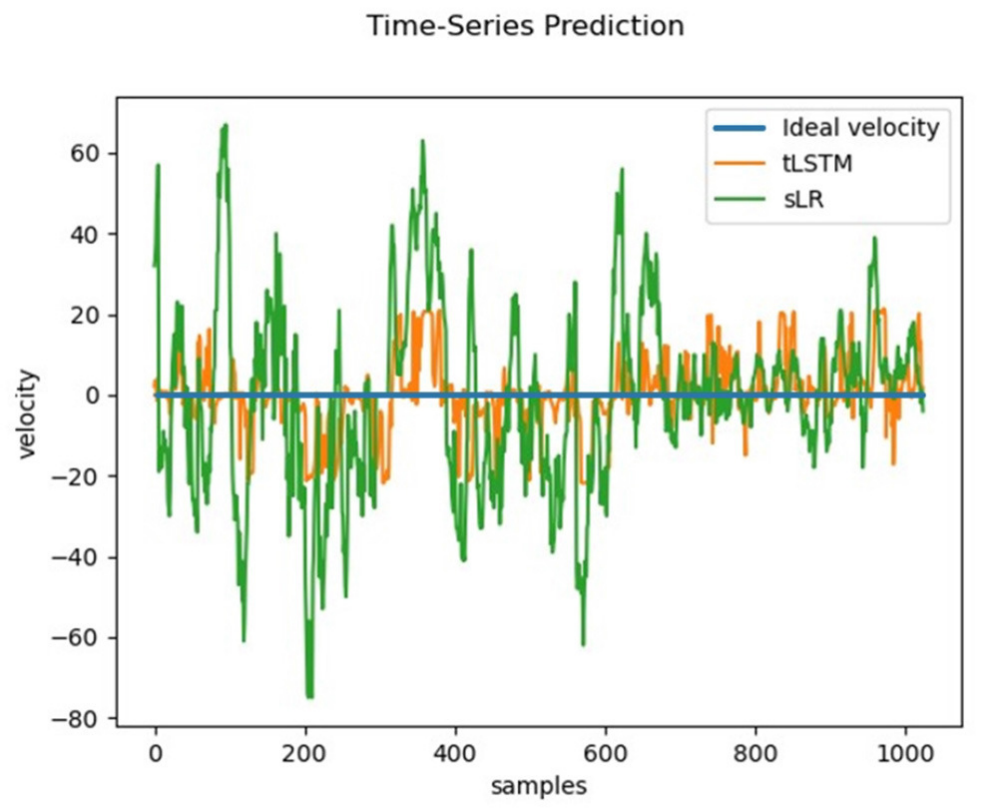
Velocity in the horizontal direction generated by tLSTM on the TESTUD dataset.

A hypothesis used in this study is that when the subject notices a large movement velocity of the cursor in the direction perpendicular to the imaginary direction, error-related signals will be generated in the EEG signals of the subject's brain and these signals will be available to correct 2D cursor's movement deviation. To test this hypothesis, the output of the tLSTM model using only error-related temporal features on the TESTUD dataset is shown in Figure 8.

The ideal velocity (blue) of samples in the horizontal direction of the TESTUD dataset is 0. The velocities generated by sLR in the horizontal direction (green) exhibit significantly larger deviations, while the velocities generated in the horizontal direction by tLSTM (orange) exhibit a smaller deviation than that of sLR, indicating that the tLSTM model is able to decode the velocity deviations from the EEG and correct them. When the actual velocity deviates from the ideal velocity, tLSTM utilizes the error related signals to suppress the deviation, hence, the actual velocity gradually tends to the ideal velocity. Therefore, the error-related temporal features can suppress the motion deviation in the non-imaginary direction.

**Figure 9:**
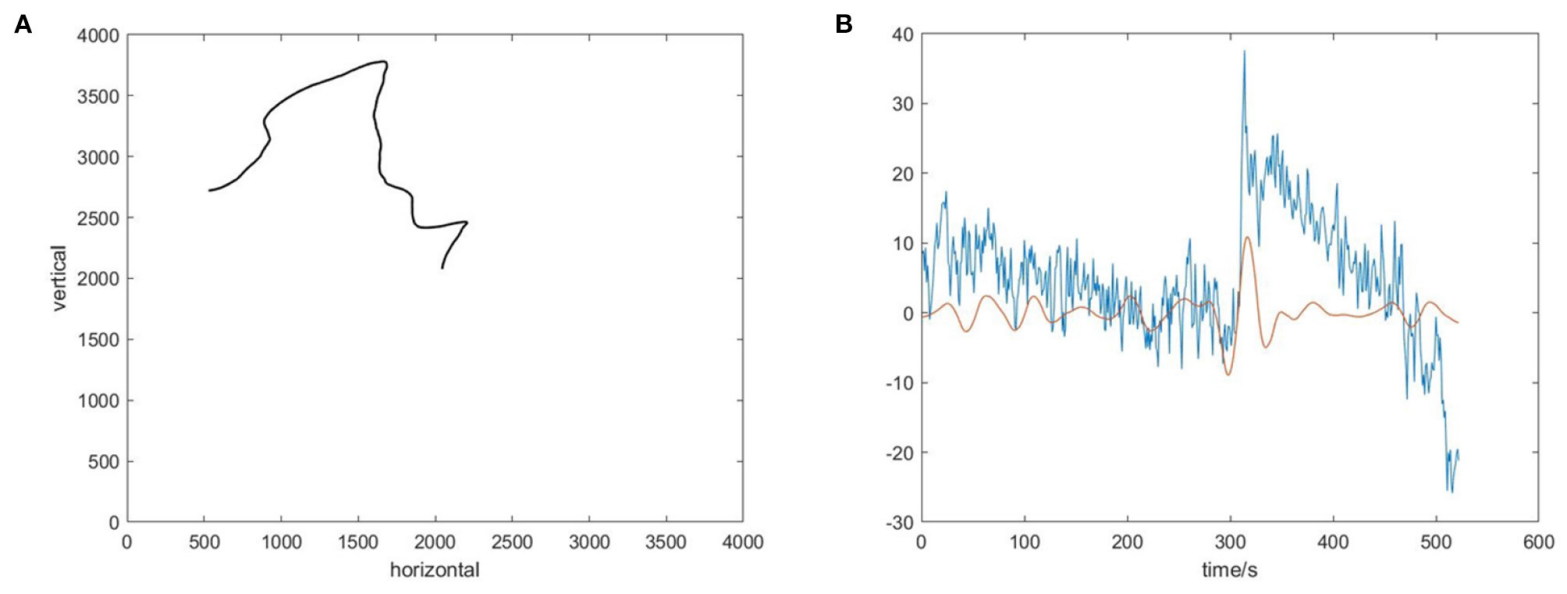
The visualization of temporal features. **(A)** The actual trajectory of the 2D cursor in one trial. **(B)** The EEG signal in the FC4 channel(blue) and the 3-7Hz wavelet transform (WT) of the EEG signal in the FC4 channel(red).

The effect of temporal features on the results generated by the model was discussed above. Let us explore why temporal features are useful. Figure 9A shows the actual cursor trajectory in one trial, and the corresponding ideal trajectory is horizontal to the left. Figure 8 shows the EEG signal recorded in the FC4 channel in this trial and the 3–7 Hz WT of the EEG signal at FC4, respectively.

In Figure 9, at about 3s, the cursor exhibits a large movement deviation in the vertical direction, corresponding to a large peak in the EEG signal at this time. This phenomenon indicates that the original EEG signal contains observed error-related information. On account of that error related signals usually show increased theta activity after the stimulus (Chavarriaga et al., 2014), we plot the 3–7 Hz time series extracted by WT of the EEG signal at FC4, which shows a large deflection at about 3s. This indicates that error-related temporal information is indeed recorded in the EEG signal when the subject observes the error, and this information could be used to effectively improve the control accuracy of the model.

### 5.3. Further Analysis for sLR and stLSTM

The sLR method (Meng et al., 2016) utilizes the EEG spectral features and combines the linear regression method to obtain the moving velocity of the cursor, and the stLSTM method utilizes the EEG spectral features + error-related temporal features and combines the LSTM network to obtain the velocities. These two methods can be understood as essentially performing spatial filtering on multi-channel EEG signals and obtaining desired filtering information. In general, there are several ways to learn such a spatial filter: (a) No Learning: using fixed *ad hoc* filters instead, such as Common Average Reference, Bipolar Derivations, and Surface Laplacian Derivations. The performance of the spatial filters obtained by this method is often not abysmal but far from optimal, there is still room for improvement. (b) Top-down: using neural-network like back-propagation/GD. This is supervised learning, with input *X*, desired outputs *y* and spectral mapping in between are all known. (c) Bottom-up: directly learning a good spatial filter for the data without the labels *y*. This is unsupervised learning, such methods contain Independent Component Analysis, Dictionary Learning, and Principal Component Analysis. (d) Performing a mixture of unsupervised and supervised learning, such as Supervised ICA, combining unsupervised pre-training with supervised fine-tuning. (e) Using direct observations: observing the spatial filter directly from data. If given a magnetic resonance scan, methods like Beamforming can be utilized. (f) Using additional assumptions: some powerful assumptions, such as the source activation in the time window of interest is jointly Gaussian-distributed, can make the problem solvable. The sLR model can be understood as a spatial filter obtained by the No Learning method. Methods in Edelman et al. (2019) and Suma et al. (2020) can be categorized into the Using direct observations method, with the prerequisite that the volume conduction model of the brain is known. The stLSTM method proposed in this article can be regarded as the Top-down method to learn a spatial filter, with the collected labeled training data to train the model in a supervised learning manner.

### 5.4. Limitations

Although it is can be seen from the previous sections that our method exhibits some performance advantages over sLR, it still has many limitations. First, our experiments were carried out offline based on a public EEG dataset mentioned in Section 2, lacking online inference results. Due to the need for the determination of parameters of stLSTM through supervised learning, the online experiment consists of two parts: the training phase and the testing phase, in the context of a certain pre-designed experimental paradigm. Therein, in the training phase, the moving direction and the moving velocity of the cursor can be predefined artificially in each trial, and the subject executes corresponding motor imagery according to some visual prompt of this trial. In the meanwhile, some trials are randomly chosen by a prefixed probability, and the moving direction or velocity of the cursor in these trials is set to be different from the visual prompt to the subject, so as to stimulate the error-related signals. The recorded EEG signals and the corresponding labels in all trials in the training phase can be utilized to train the model. In the real-time testing phase, the trained stLSTM model is able to decode the subject's EEG signal by combining the spectral and temporal features and converting it into the control velocity of the cursor.

Second, the tasks of the EEG dataset used in this article are limited to simple tasks in which the trajectories are composed of movements in horizontal and vertical directions. Some more complex experimental paradigms can be devised in subsequent studies. Finally, different from traditional machine learning methods, the proposed decoding model adopts a deep neural network backbone model based on LSTM, which shows the inherent disadvantage of poor interpretability, i.e., it is difficult to determine the physical significance of each parameter in the model. Therefore, we will explore these limitations and conduct more experiments in the future.

## 6. Conclusion

This article proposes a novel EEG decoding framework to control 2D cursor movement. Both spectral and temporal features are used to improve the robustness and accuracy of the cursor control. Specifically, spectral features are extracted by an AR model; error-related temporal features are extracted by convolving the original EEG signals with a wavelet function, aiming to correct movement biases that do not conform to the user's intention. Finally, the concatenated feature vectors are used as the input to be fed into the stLSTM, and *L*_*stLSTM*_ is used as the optimization function of the model to generate the vertical and horizontal velocity. The movement trajectory of the 2D cursor can be obtained by integrating the velocities separately. The performance of the proposed method has been evaluated on a public EEG dataset. The proposed method exhibits promising results in terms of metric RMSE, ACC, MAR. Series of experimental results confirm that our proposed method can be regarded as a control system of the 2D cursor based on a noninvasive electroencephalogram, suppressing the velocity in the non-imaginary direction and improving the accuracy of the cursor's movement.

## Data Availability Statement

The original contributions presented in the study are included in the article/supplementary material, further inquiries can be directed to the corresponding author.

## Author Contributions

KP and YG designed research. KP, LL, SL, and ZY performed research. KP, YG, and LL wrote the manuscript. LZ edited the manuscript. All authors reviewed the manuscript, contributed to the article, and approved the submitted version.

## Funding

This study was funded by the Natural Science Foundation of Beijing Municipality, China (Grant No. 4202040), the National Natural Science Foundation of China (Grant No. 61876015), and the Science and Technology Innovation 2030 Major Program of China (Grant No. 2018AAA001400).

## Conflict of Interest

The authors declare that the research was conducted in the absence of any commercial or financial relationships that could be construed as a potential conflict of interest.

## Publisher's Note

All claims expressed in this article are solely those of the authors and do not necessarily represent those of their affiliated organizations, or those of the publisher, the editors and the reviewers. Any product that may be evaluated in this article, or claim that may be made by its manufacturer, is not guaranteed or endorsed by the publisher.
